# Hydroxysteroid Sulfotransferase SULT2B1b Promotes Hepatocellular Carcinoma Cells Proliferation *In Vitro* and *In Vivo*

**DOI:** 10.1371/journal.pone.0060853

**Published:** 2013-04-11

**Authors:** Xiaoming Yang, Yali Xu, Fenghua Guo, Yanxia Ning, Xiuling Zhi, Lianhua Yin, Xiaobo Li

**Affiliations:** 1 Department of Physiology and Pathophysiology, Fudan University Shanghai Medical College, Shanghai, China; 2 Ningxia Medical University, Yinchuan, Ningxia, China; 3 General Surgery, Hua’shan Hospital, Fudan University Shanghai Medical College, Shanghai, China; Rush University Medical Center, United States of America

## Abstract

Hydroxysteroid sulfotransferase 2B1b (SULT2B1b) is highly selective for the addition of sulfate groups to 3β-hydroxysteroids. Although previous reports have suggested that SULT2B1b is correlated with cell proliferation of hepatocytes, the relationship between SULT2B1b and the malignant phenotype of hepatocarcinoma cells was not clear. In the present study, we found that SULT2B1 was comparatively higher in the human hepatocarcinoma tumorous tissues than their adjacent tissues. Besides, SULT2B1b overexpression promoted the growth of the mouse hepatocarcinoma cell line Hepa1-6, while Lentivirus-mediated SULT2B1b interference inhibited growth as assessed by the CCK-8 assay. Likewise, inhibition of SULT2B1b expression induced cell-cycle arrest and apoptosis in Hepa1-6 cells by upregulating the expression of FAS, downregulating the expression of cyclinB1, BCL2 and MYC *in vitro* and *in vivo* at both the transcript and protein levels. Knock-down of SULT2B1b expression significantly suppressed tumor growth in nude mouse xenografts. Moreover, proliferation rates and SULT2B1b expression were highly correlated in the human hepatocarcinoma cell lines Huh-7, Hep3B, SMMC-7721 and BEL-7402 cells. Knock-down of SULT2B1b inhibited cell growth and cyclinB1 levels in human hepatocarcinoma cells and suppressed xenograft growth *in vivo*. In conclusion, SULT2B1b expression promotes proliferation of hepatocellular carcinoma cells *in vitro* and *in vivo*, which may contribute to the progression of HCC.

## Introduction

Hepatocellular carcinoma (HCC) is a major cause of cancer mortality worldwide and is often associated with a poor prognosis. Although the pathogenesis of HCC has been studied at depth, the exact mechanisms and pathways leading to its development are still unclear [1], [2]. A growing body of literature has demonstrated that aberrant lipid metabolic abnormalities may increase the susceptibility of the liver to tumorigenesis [3], [4]. As the liver is a major source of cholesterol biosythesis and metabolism, elevated levels of cholesterol are oxygenated to oxysterols by cytochrome P450 or by non-enzymatic reactions involving reactive oxygen and nitrogen species. Numerous studies have shown that oxysterols contribute to the initiation and progression of various types of cancer, including cancers of the colon, lung, breast and bile ducts [5], [6], [7], [8]. To date, the relationship between oxysterol and hepatocellular carcinoma remains unknown.

The hydroxysteroid sulfotransferase 2B1b (SULT2B1b) had previously been characterized as a cholesterol and oxysterols sulfotransferase. SULT2B1b was encoded by *SULT2B1* gene. SULT2B1 has two isoforms, SULT2B1a and SULT2B1b, resulting from alternative splicing of the *SULT2B1* gene [9]. SULT2B1b is highly selective for the sulfation of 3β-hydroxysteroids such as cholesterol, oxysterols, DHEA, D^5^-Adiol, and 5a-Androstane-3β-17β-diol (Anstane-diol) and pregnenolone [10]. SULT2B1b is also responsible for sulfating 25-hydroxychoelsterol into 5-Cholesten-3β-25-diol-3-sulfate (25HC3S), which is a novel regulatory oxysterol [11]. Previous reports have shown that SULT2B1b is expressed in a variety of hormone-responsive tissues including the ovary, uterus, placenta, prostate and breast [12]. In addition, SULT2B1 expression is significantly altered in prostate, breast, and endometrial tumors relative to normal tissues [13], [14], [15]. However, there are few reports describing the role of SULT2B1 in the progression of hepatocellular carcinoma.

Sasso *et al*. [16] previously reported that SULT2B1 was transcriptionally up-regulated during liver regeneration accompanied with activation of liver X receptor (LXR) in a mouse model of partial hepatectomy (PH). Similarly, Zhang *et al*. [17] has demonstrated the expression of SULT2B1b transcript and protein in hepatocytes using real-time quantitative PCR (qPCR) and Western-blot analysis. Additionally, overexpression of SULT2B1b by adenovirus transduction was found to dramatically promote liver proliferation. This evidence suggests that SULT2B1b functionally enhances hepatocellular proliferation, especially in HCC cells where cell proliferation is left unchecked. We hypothesized that SULT2B1b may enhance proliferation in HCC cells, contributing to the progression of HCC.

In the present study, the data demonstrates that overexpression of SULT2B1b in hepatocellular carcinoma cells promotes, while SULT2B1b knock-down inhibits proliferation both *in vivo* and *in vitro*, suggesting a role for SULT2B1b in the development of HCC.

## Materials and Methods

### Clinical Samples Collection

Six cases of human hepatocarcinoma tissue samples were obtained from General Surgery of Fudan University affiliated Hua’shan hospital. Tumor tissue and para-tumor tissue were involved in each case.

### Ethics Statement

All experimental procedures involving animals were approved by IACUC committee of Fudan University (20120302-014) and were performed in accordance with the institutional ethical guidelines for animal.

### Cell Culture

Mouse and human hepatocarcinoma cell lines, Hepa1-6, Huh-7 and Hep3B, were obtained from the American Type Culture Collection. The human hepatocarcinoma cell line SMMC-7721 and BEL-7402 cells were purchased from the Type Culture Collection of the Chinese Academy of Sciences, Shanghai, China. Human glioblastoma cells, U87, were obtained from School of Life Sci., Fudan University. All of the cells were maintained in Dulbecco’s modified Eagle’s medium supplemented with 10% fetal calf serum (Gibco, Carlsbad, CA, USA). The cells were incubated in 5% CO_2_ at 37°C and 95% humidity.

### Immunofluorescence

Treated cells were seeded into 24-well plates in 10% FBS-containing medium. Cells were washed with PBS and fixed with 4% paraformaldehyde for 10 min at room temperature and then rinsed three times with PBS. Cells were then permeabilized in PBS containing 0.1% Triton X-100 and washed with PBST before blocking with 5% goat serum for 30 min at 37°C. For immunostaining, cells were incubated with anti-SULT2B1 (Abcam, Cambridge, MA, USA, 1∶100) for 1 h at 37°C followed by incubation with FITC conjugated goat anti-rabbit secondary antibody (1∶200) for 40 min at 37°C. Cells were then washed with PBST twice and nuclei were counterstained with DAPI for 5 min. Cell morphology was visualized with a Zeiss LSM 510 Meta confocal microscope.

### Modulation of SULTB1 Expression using Cell Transduction with siSULT2B1 Lentivirus or SULT2B1b Adenovirus

Mouse SULT2B1 small interfering RNA (mSULT2B1-RNAi-LV, target sequence, CAGTGTTTACCGAGAGCAAAT; TU = 1.5×10^9^/mL), human SULT2B1 small interfering RNA (hSULT2B1-RNAi-LV, target sequence: GGGACTTCCTCAAAGGCGA; TU = 3×10^8^/mL) were prepared by Genechem corporation (Shanghai, China). Lenti virus-mediated GFP (NC-GFP-LV, TU = 2×10^9^/mL) and RFP (NC-RFP-LV, TU = 1×10^9^/mL) were used as a negative controls. The recombinant adenovirus encoding human SULT2B1b vectors (Ad-SULT2B1b, TU = 6×10^8^/mL) was prepared as described previously [18], Adenovirus-mediated negative control (Ad-EGFP, TU = 6×10^8^/mL) was used as a non-specific control vector.

Mouse and human hepatocarcinoma cell lines, Hepa1-6 and BEL-7402, were plated into 12-well plates (4×10^4^ cells per well), and then infected with lentivirus-mediated SULT2B1 RNAi and hSULT2B1 RNAi vectors in serum-free medium at a multiplicity of infection (MOI) of 100 respectively, using polybrene (5 µg/µL) reagent to increase the efficiency of infection according to the manufacturer’s protocol, NC-GFP-LV and NC-RFP-LV were used negative control respectively. After 12 hours incubation, the medium was changed to DMEM supplemented with 10% FBS. For adenovirus mediated SULT2B1b infection, Hepa1-6 cells were plated on 60 mm dish (5×10^5^ cells), then transfected with SULT2B1b overexpression vectors Ad-SULT2B1b in serum-free media with indicated MOI, Ad-EGFP was used as a negative control, 2 hours later, the medium was changed to DMEM supplemented with 10% FBS. And then the cells were incubated for another 48 h before proceeding with experiments.

### Transient Transfection

Human hepatocarcinoma cell lines, SMMC-7721 and BEL-7402, were cultured in 6-well plates (3×10^5^ cells per well) a day before transfection. Cells were transiently transfected the human SULT2B1 (Target sequence: GCTCCAAGGCCAAGGTGAT) and SULT2B1b-specific small interfering RNA (Target sequence: CGGAAATCAGCCAGAAGTT) by Lipofectamine 2000 according to the manufacturer’s instructions, control-siRNA was used as negative control. After 48 h, cells were collected for total RNA and protein extraction.

### Cell Proliferation Assay

Cell proliferation was assessed by the cell counting kit-8 (CCK-8) assay according to the manufacturer’s protocol (Dojindo Laboratories, Gaithersburg, MD, USA). Cells in a 96-well plate were incubated with CCK-8 solutions for 1 h at 37°C. Absorbance of each well was quantified at 450 nm by the Tecan Infinite 2000 Microplate Reader.

### Flow Cytometry

Cell cycle analysis was performed using ethanol-fixed cells stained with propidium iodide in buffer containing RNaseA. The DNA content was assessed using a FACS Calibur Flow Cytometer (Becton-Dickinson, San Jose, CA, USA). Apoptotic cells were assessed following the manufacturer’s protocol (Becton-Dickinson). Briefly, treated cells were collected, washed twice with ice-cold PBS, resuspended in binding buffer at a concentration of 1×10^6^ cells/mL, incubated with Annexin V-PE (Phycoerythrin) and 7-ADD (7-Amino-actinomycin) for 15 min at room temperature, and then analyzed by ﬂow cytometry within 1 hr.

### Determination of SULT2B1 Isoforms by RT-PCR Analysis

For detection of SULTB1 isoforms, total RNA isolated from human hepatocarcinoma cells (SMMC-7721, BEL-7402, Huh-7 and Hep3B cells), and mouse Hepa1-6 cells transduced with NC-GFP-LV or mSULT2B1-RNAi-LV. Reverse transcription (RT) was performed using RevertAid™ First Strand cDNA Synthesis Kit (Fermentas) according to the manufacturer's instructions. PCR was performed using universal primer mix Tag and human and mouse SULT2B1 isoform-specific primers found in Table S1 and previously described [19].

PCR conditions for mouse SULT2B1 isoforms were 95°C for 5 min, followed by 35 cycles of 94°C for 15 sec, 55°C for 15 sec, and 72°C for 30 sec, using mouse brain tissue as mouse SULT2B1a positive control. The PCR conditions for human SULT2B1 isoforms was the same as above, but with an annealing temperature of 60°C, using U87 cell as human SULT2B1a positive control. β-actin and GADPH were used as internal controls for mouse and human hepatocarcinoma cell lines, respectively. The PCR products were visualized on a 2% agarose gel containing 5 mg/ml ethidium bromide. Expected sizes of mouse SULT2B1a, mouse SULT2B1b, human SULT2B1a, human SULT2B1b PCR products were 341 bp, 300 bp, 123 bp and 122 bp respectively. The fragments were further sequenced by Sangon Biotech Co., Ltd (Shanghai, China).

### Real-time Quantitative PCR (qPCR)

Total RNA was extracted using the TRIZOL reagent (Invitrogen, USA) according to the supplier's instructions. Two micrograms of total RNA was used for first-strand cDNA synthesis as recommended by the manufacturer (Fermentas). Specific mRNA levels were determined by qPCR as previously described [20]. Specific primer pairs in the experiment were listed in Table 1, and referenced in the primer bank [21].

**Table 1:** Primer sets used for qPCR.

Gene	GenBank Number	Sense Primer (5' – 3')	Antisense Primer (5' – 3')
mouse SULT2B1b	NM_017465	GTGGAGCTCGTCTGAGAAAAATGTTTCCG	TTGAAGGCGCTTATGATGGTCTCGC
mouse FAS	NM_001146708.1	GATCTGGGCTGTCCTGCCTCT	TTCACGAACCCGCCTCCTC
mouse BCL2	NM_009741.3	AGCGTCAACAGGGAGATG	CCAGGTATGCACCCAGAG
mouse MYC	NM_001177352.1	ACCTCGTCCGATTCCAC	CTTCCTCATCTTCTTGCTC
mouse cyclin B1	NM_172301.3	GCGTGTGCCTGTGACAGTTA	CCTAGCGTTTTTGCTTCCCTT
mouse β-actin	NM_007393.3	GGCTGTATTCCCCTCCATCG	CCAGTTGGTAACAATGCCATGT
human SULT2B1	NM_177973.1	AGTTTGGCTCCTGGTTGG	GAGGCAGCAGCGTGTAGTT
human GADPH	NM_002046	AACGGATTTGGTCGTATTG	GGAAGATGGTGATGGGATT
human SULT2B1b	NM_177973.1	ATGACATCTCGGAAATCAGCCA	GCACATCTTGGGTGTTCTCCG
human cyclinB1	NM_031966.3	GAGGAAGAGCAAGCAGTC	TAGCCAGTCAATTAGGATG

### Western-blot Assay

After the indicated treatment, cells were harvested in radioimmune precipitation assay (RIPA) lysis buffer. Proteins were separated by 10% SDS-PAGE and transferred to PVDF using standard techniques. Immunoblots were probed with anti-SULT2B1 (Abcam, Cambridge, MA, USA, 1∶500), anti-FAS (Proteintech Group, Inc. Chicago, USA, 1∶500), anti-cyclinD1, anti-cyclinB1 (Cell Signaling Technology, Inc. Danvers, MA,USA, 1∶200 and 1∶500), anti-BCL2, anti-MYC, anti-β-actin, and anti-tubulin (Bioword, Louis Park, MN 55416, USA, 1∶500). β-actin or tubulin was used as a loading control. The band intensities were quantified by densitometry using the software of GIS (Bio-Tanon, Shanghai, China).

### Detection of SULT2B1 Sulfotransferase Activity in Hepa1-6 Cells

Hepa1-6 cells treated with GFP-LV or SULT2B1-RNAi-LV were harvested in 10 mM KPO_4_ buffer (pH 7.4). SULT2B1 activity was measured in 100 µL of 25 mM Tris-Cl (pH 7.2) buffer containing 0.02 nmol of [^3^H]-cholesterol (1 µCi) dissolved in 3 µL of ethanol, 100 µg of total protein, 5 mM MgCl_2_, 8 mM DTT, 100 µM 3′-phosphoadenosyl 5′-phosphosulfate (PAPS) at 37°C for 1 h. Lipids were extracted with 3.3 volumes of chloroform-methanol (1∶1, v/v). The radioactivity counts in methanol-water-soluble phase and chloroform phase were determined by liquid scintillation counting. The difference of SULT2B1 activity between GFPLV and SULT2B1-RNAi-LV treated cells was calculated by the ratio of methanol-water-soluble counts to the sum of chloroform/methanol-water-soluble counts.

The SULT2B1 activity in Hepa-16 cells was also detected by the conversion rate of [^3^H] cholesterol to [^3^H] methanol-water-soluble counts by adding [^3^H] cholesterol to the cells. 5×10^4^ Hepa1-6 cells treated with GFP-LV or SULT2B1-RNAi-LV were cultured in 24-well plates with 500 µL culture medium. After attachment, cells were incubated with 20 nM [^3^H] cholesterol (53 Ci/mol; Perkin Elmer, San Jose, CA, USA) and 50 µM PAPS for the sulfation assay. After 16 h, the attached cells were washed with PBS, and then harvested in 50 µL of PBS. Conversion of [^3^H] cholesterol to [^3^H] methanol-water-soluble products was determined by scintillation counting after extraction with 3.3 volumes of chloroform-methanol (1∶1, v/v) from cells. The rates of cholesterol sulfation following GFP-LV or SULT2B1-RNAi-LV infection were calculated as the ratio of [^3^H] methanol water-soluble counts to the sum of chloroform/methanol water-soluble counts.

### Murine Xenograft Model for Tumorigenicity Assay

Six-week old male BALB/c-nude mice were used for experimental tumorigenicity assays.Mouse Hepa1-6 cells (1.5×10^6^) that were transduced with NC-GFP-LV or mSULT2B1-RNAi-LV and human BEL-7402 cells (1.5×10^6^) transduced with NC-RFP-LV or hSULT2B1-RNAi-LV were injected subcutaneously into the subaxillary space of each mouse. Mice were weighed and the tumor width (W) and length (L) were measured every three days. Tumor volume was estimated according to the standard formula 1/2×L×W^2^. Observation continued until day 18∼21. Tumors were excised from the animals, and then frozen in liquid nitrogen and stored at −80°C.

### Statistical Analysis

For all experiments, each treatment condition was conducted in triplicate and repeated at least three times. The results of multiple observations are presented as the mean ± standard error of the mean of at least three separate experiments. Statistical significance was determined by one-way ANOVA for multiple comparisons or by independent-sample Student’s t-test, Pearson correlation and simple linear regression analysis using SPSS 11.5 software. *P*<0.05 was considered to be significant.

## Results

### SULT2B1b Promoted the Growth of Hepa1-6 Cells *in vitro*

SULT2B1 expression was detected in C57BL/6 mouse liver, primary mouse hepatocytes purified from C57BL/6 mice liver as described in [22], and in the mouse hepatocarcinoma cell line, Hepa1-6, by Western-blot assay. As shown in Figure S1A, the SULT2B1 protein level in Hepa1-6 cells was much higher than normal mouse liver tissue and primary mouse hepatocytes. Figure S1B demonstrates SULT2B1 localization in Hepa1-6 cells by immunofluorescence. SULT2B1 isoforms were detected in Hepa1-6 cells transduced with NC-GFPLV or SULT2B1-RNAi-LV by RT-PCR (Figure S1C). While SULT2B1a expression was absent, SULT2B1b was detected.

**Figure 1:**
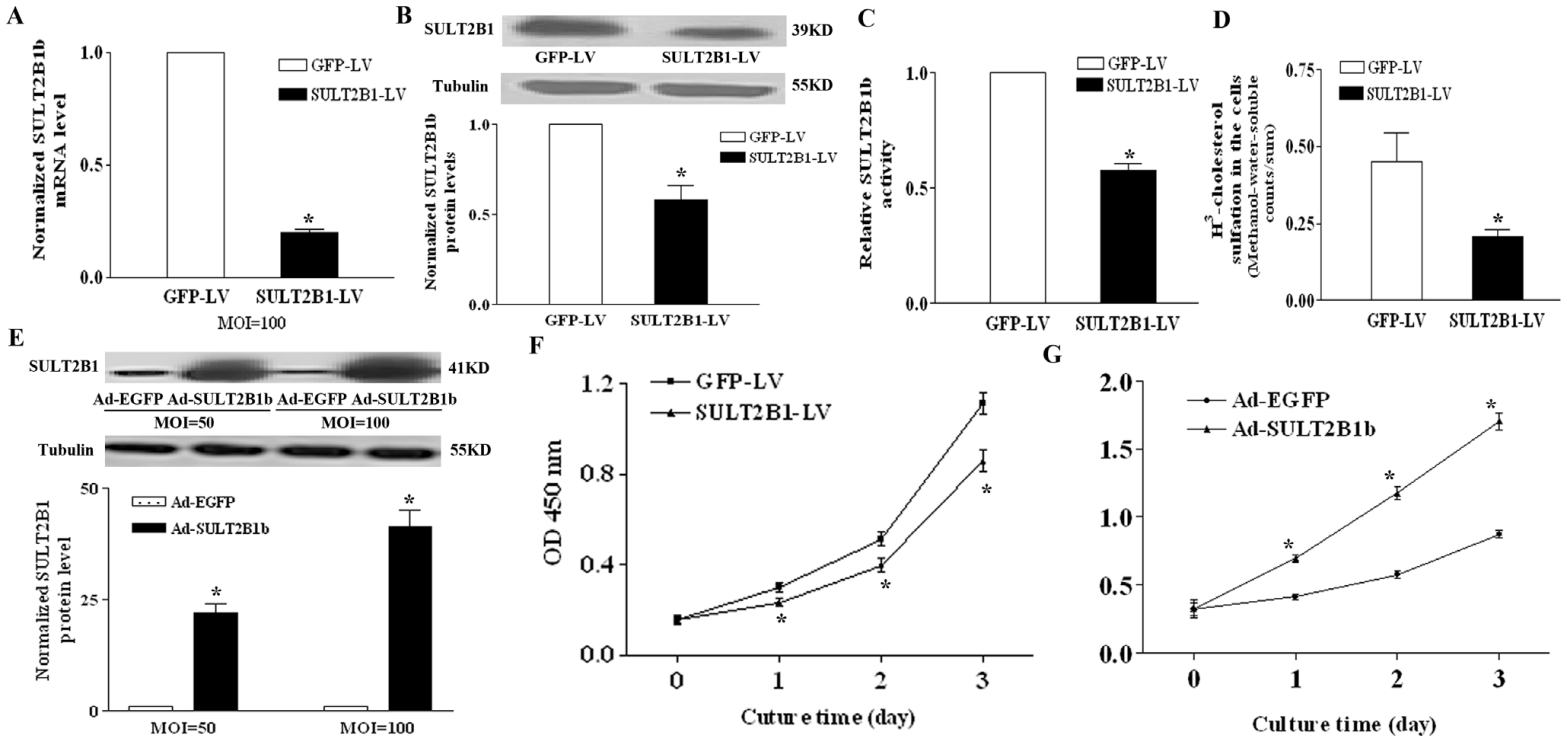
SULT2B1b promotes the growth of mouse hepatocarcinoma cells *in vitro*. The mRNA (A) and protein (B) levels of SULT2B1 in transduced Hepa1-6 cells were detected by qPCR and western blot analysis respectively. SULT2B1 sulfotransferase activity in transduced Hepa1-6 cells was detected by sulfotransferase assay *in vitro* (C) or by measuring the conversion rate of [^3^H] cholesterol to [^3^H] methanol-water-soluble counts by adding [^3^H] cholesterol to live cells (D). SULT2B1b protein expression in Hepa1-6 cells transduced with Ad-EGFP or Ad-SULT2B1b (E). CCK-8 assay of the growth curves of Hepa1-6 cells transduced with SULT2B1-RNAi-LV, Ad-SULT2B1b, or vector controls (F,G). *represents *P*<0.05 vs. Ad-GFP group or NC-GFP-LV group.

As shown in Fig. 1A, qPCR analysis revealed that lentivirus-mediated SULT2B1b siRNA decreased the mRNA level of SULT2B1b by 81.2% in comparison with NC-GFP-LV at a multiplicity of infection (MOI) of 100. SULT2B1 protein level in Hepa1-6 cells decreased accordingly after SULT2B1-RNAi-LV treatment (Fig. 1B). The SULT2B1 sulfotransferase activity also decreased with SULT2B1 knock-down based on the SULT2B1 activity assay *in vitro* (Fig. 1C). The reduced SULT2B1 sulfotransferase activity in Hepa-16 cells treated by SULT2B1-RNAi-LV was also confirmed by the decreased conversion rate of [^3^H]-cholesterol to [^3^H]-methanol-water-soluble counts (Fig. 1D). SULT2B1 protein level in Hepa1-6 cells increased significantly with over-expression of SULT2B1b (Fig. 1E). Using CCK-8 assay, the effect of SULT2B1b interference and SULT2B1b overexpression on the growth of hepatocarcinoma cells was assessed. SULT2B1-RNAi-LV inhibited the growth of Hepa1-6 cells compared to control GFP–LV (Fig. 1F), while overexpression of SULT2B1b promoted cell growth compared with the control Ad-EGFP (Fig. 1G).

### Knock-down of SULT2B1b Induced Cell-cycle Arrest and Apoptosis in Hepa1-6 Cells

**Figure 2:**
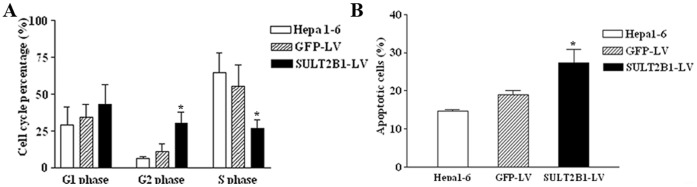
Knock-down of SULT2B1b induced cell-cycle arrest and apoptosis in Hepa1-6 cells. (A) Flow cytometry analysis of the percentage of cells in different phases of the cell cycle in untransduced Hepa1-6 cells or Hepa1-6 cells transduced with vector control or SULT2B1-RNAi-LV analyzed with propidium iodide staining. (B) Flow cytometry analysis of the percentage of apoptotic cells in untreated Hepa1-6 cells or Hepa1-6 cells treated with vector control or SULT2B1-RNAi-LV as analyzed by Annexin V-APC and 7-ADD staining. The data represents the mean of three independent experiments ± S.D. *represents *P*<0.05 vs. NC-GFP-LV group.

The cell cycle and cell apoptosis were analyzed to elucidate the mechanisms underlying knock-down of SULT2B1b induced growth inhibition. Compared with NC-GFP-LV and non-transduced cells, more SULT2B1-RNAi-LV cells were in the G_2_ phase, while fewer cells were in the S phase. No differences in cell numbers were observed in the G_1_/G_0_ phase (Fig. 2A). These results suggest that SULT2B1b knock-down might block the G_2_/M transition. Additionally, apoptosis was significantly increased in Hepa1-6 siSULT2B1b cells (Fig. 2B).

### Effect of SULT2B1b Interference or Overexpression on the Expression of FAS, BCL2 and MYC in Hepa1-6 Cells

**Figure 3:**
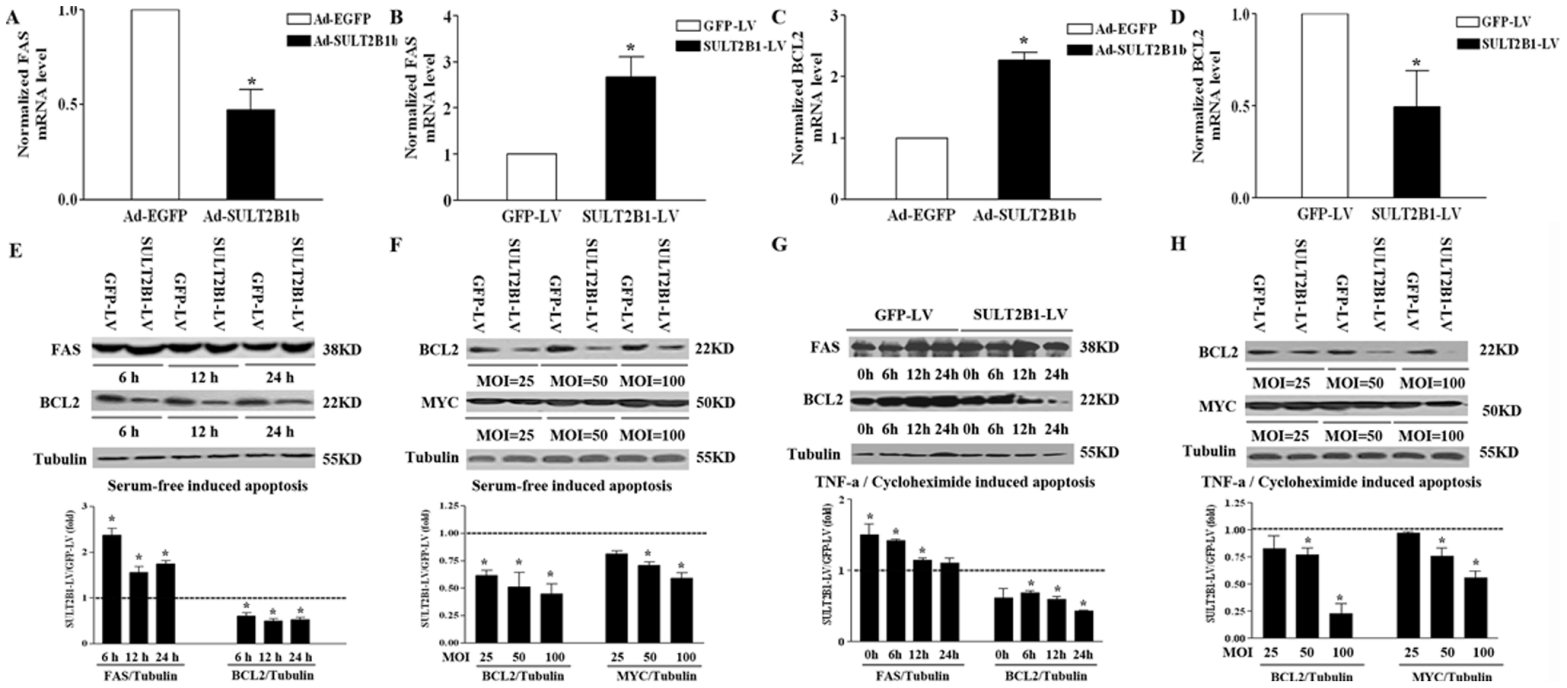
The effect of SULT2B1b interference or overexpression on the expression of FAS, BCL2 and MYC in Hepa1-6 cells. The mRNA levels of FAS and BCL2 following SULT2B1b overexpression (A, C) and SULT2B1b inhibition (B, D) were analyzed by qPCR. Western blot analysis of: (E) FAS and BCL2 protein levels in Hepa1-6 cells treated with NC-GFP-LV or SULT2B1-RNAi-LV upon serum starvation at 6 h, 12 h and 24 h. (F) The protein levels of BCL2 and MYC in Hepa1-6 cells treated with NC-GFP-LV or SULT2B1-RNAi-LV at different multiplicity of infections (MOI = 25, 50, 100) and cultured in serum-deprived medium for 24 h. (G) FAS and BCL2 protein levels in NC-GFP-LV or SULT2B1-RNAi-LV Hepa1-6 cells cultured in serum-free medium containing 10 ng/mL TNF-α and 10 µg/mL CHX for the indicated times. (H) The BCL2 and MYC protein levels in Hepa1-6 cells infected with NC-GFP-LV and SULT2B1-RNAi-LV at MOI of 25, 50, 100 and treated with 10 ng/mL TNF-α and 10 µg/mL CHX. *represents *P*<0.05 vs. Ad-GFP group or NC-GFP-LV group.

The expression of proliferation and apoptosis related genes was evaluated. To identify important signaling pathways affected by SULT2B1b, the mRNA expression of the pro-apoptotic factor FAS, and the anti-apoptotic factor, BCL2, was measured. Overexpression SULT2B1b, signifcantly decreased the mRNA level of the pro-apoptotic factor, FAS (Fig. 3A), while the expression of the anti-apoptotic factor, BCL2, was upregulated (Fig. 3C). Similarly, knock-down of SULT2B1b significantly increased FAS mRNA level (Fig. 3B), but inhibited BCL2 mRNA expression (Fig. 3D).

The protein expression of FAS, BCL2 and MYC was evaluated in NC-GFP-LV and SULT2B1-RNAi-LV Hepa1-6 cells that were serum-deprived or were treated with a combination of tumor necrosis factor-a (TNF-α, 10 ng/mL) and cycloheximide (CHX, 10 µg/mL). As seen in Fig. 3, both serum deprivation (Fig. 3E, F) and TNF-α/CHX treatment (Fig. 3G, H) reduced BCL2 and MYC protein levels, while FAS protein levels were increased.

### Analysis of Cell Cycle Arrest with SULT2B1 Knock-down in Hepa1-6 Cells

**Figure 4:**
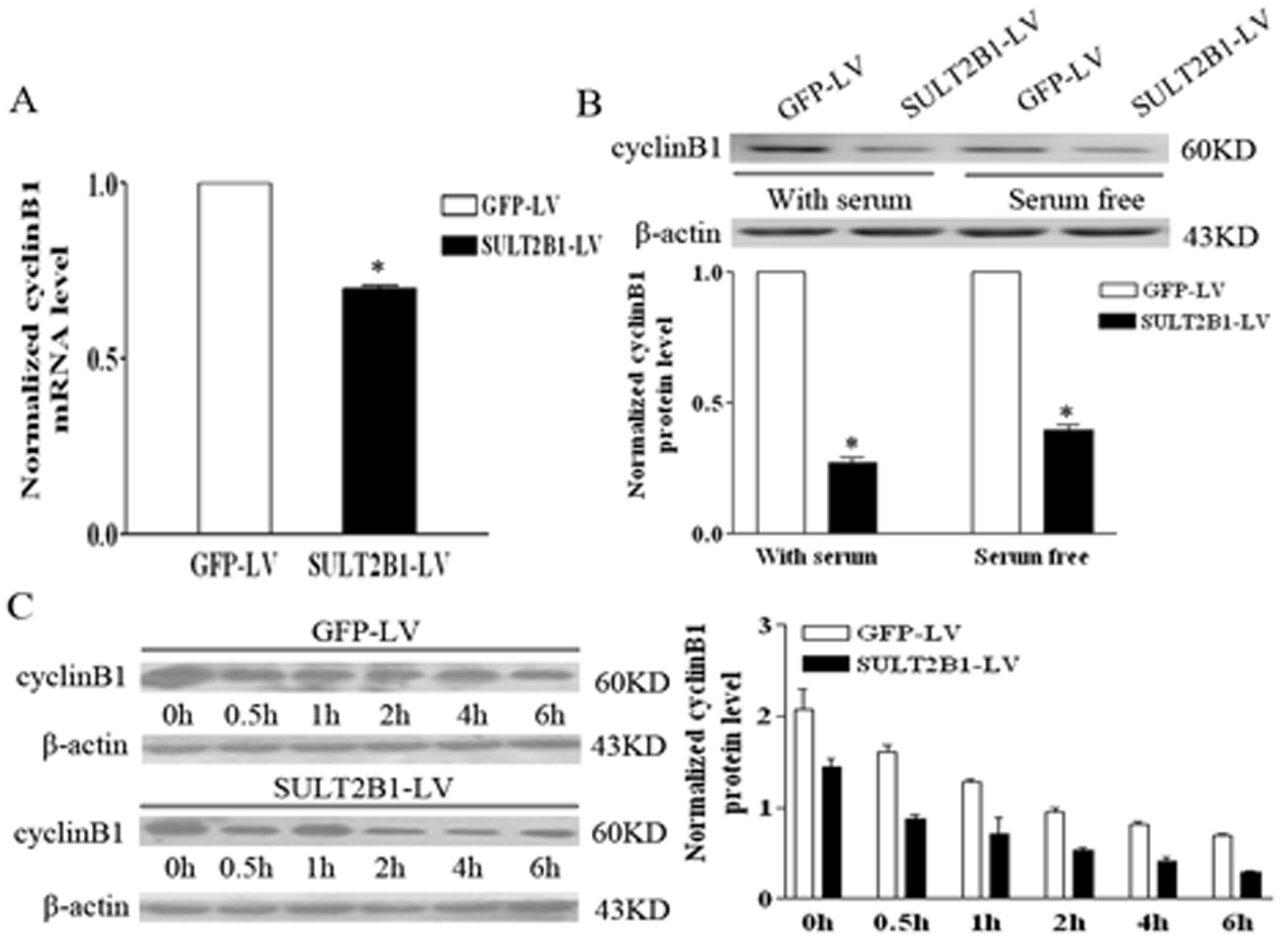
The effects of SULT2B1b interference on the expression and stability of cyclinB1 in Hepa1-6 cells. The mRNA (A) and protein (B) levels of cyclinB1 were analyzed by qPCR and Western-blot, respectively. (C) The stability of cyclinB1 protein in NC-GFP-LV or SULT2B1-RNAi-LV Hepa1-6 cells was determined with 100 µg/ml CHX treatment. *represents *P*<0.05 vs. NC-GFP-LV group.

To elucidate the mechanisms of cell-cycle arrest induced by SULT2B1b interference that presented in Fig. 4, we analyzed the expression of cyclinD1 and cyclinB1 by qPCR and Western-blot assays. Both the mRNA and protein levels of cyclinD1 presented no significant difference between siSULT2B1 and control Hepa1-6 cells (data not shown). However, as shown in Fig. 4A, cyclinB1 mRNA expression was 25% lower in SULT2B1-RNAi-LV cells compared to NC-GFP-LV control cells.

Furthermore, cyclinB1 protein levels also decreased significantly in SULT2B1-RNAi-LV cells both in 10% FBS medium and serum-free medium compared to the NC-GFP-LV group (Fig. 4B). Because the protein level of cyclinB1 can be regulated by ubiquitination and subsequent proteasomal degradation, we checked the stability of cyclinB1 protein by adding 100 µg/ml of CHX into both SULT2B1-RNAi-LV and NC-GFP-LV treated Hepa1-6 cells (Fig. 4C). The results demonstrate that the rate of cyclinB1 degradation was much faster in SULT2B1-RNAi-LV treated cells than in NC-GFP-LV treated cells.

### Knock-down of SULT2B1 in Hepa1-6 Cells Suppressed Tumorigenesis *in vivo*

**Figure 5:**
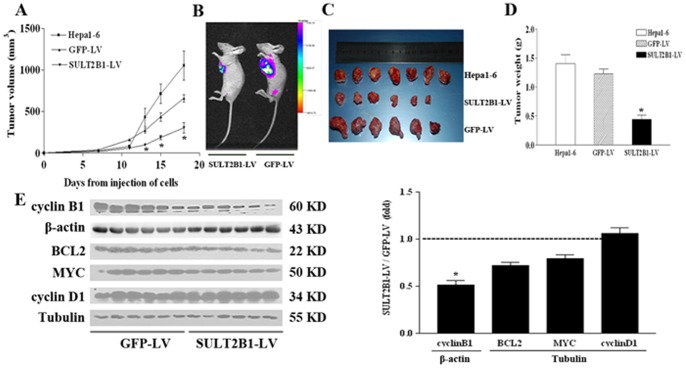
Knock-down of SULT2B1b suppressed tumorigenesis *in vivo*. 1.5×10^6^ NC-GFP-LV or SULT2B1-RNAi-LV Hepa1-6 cells were injected subcutaneously into the subaxillary space of each nude mouse. (A) The growth curve of xenografts. (B) Representative fluorescence images of xenografts. (C) Images of dissected tumors. (D) The weights of dissected tumors. (E) Western-blot analysis of the protein levels of the cell growth-associated genes, BCL2, MYC, cyclinD1, and cyclinB1 in dissected tumors. The quantification of protein levels of the above genes is shown to the right. *represents *P*<0.05 vs. NC-GFP-LV group.

We further analyzed the effect of SULT2B1 inhibition on tumorigenesis in a Hepa1-6 xenograft model. Knock-down of SULT2B1 significantly suppressed tumor growth *in vivo* as compared with NC-GFP-LV (Fig. 5A). Representative fluorescence images of xenografts confirmed these results (Fig. 5B). The tumor size and tumor weight of xenografts from siSULT2B1 cells was significantly smaller than xenografts from the GFP-LV control cells or untransduced cells (Fig. 5C, D). Furthermore, the expression of the apoptotic and proliferation genes, BCL2, MYC, cyclinD1, and cyclinB1 were chosen for further analysis. In tumor xenografts of SULT2B1-RNAi-LV cells, cyclinB1, MYC and BCL2 protein levels decreased, while no significantly differences in cyclinD1 protein levels was observed between the two groups (Fig. 5E).

### SULT2B1b Expression Correlated with Human Hepatocarcinoma Cell Proliferation

**Figure 6:**
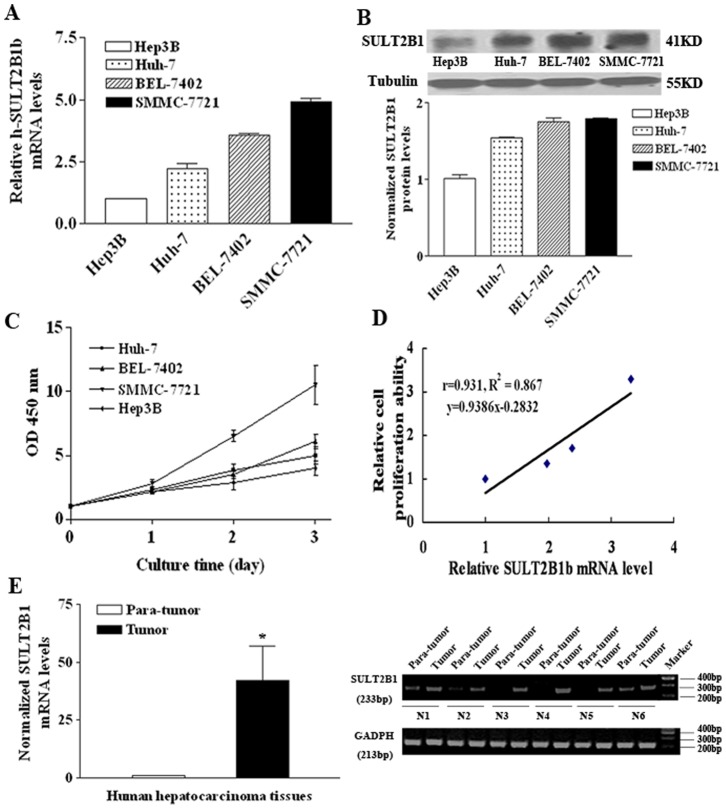
SULT2B1 promoted the growth of human hepatocarcinoma cells *in vitro*. Endogenous expression of SULT2B1b mRNA (A) and protein levels (B) was measured by qPCR and Western blot assay, respectively. (C) Proliferation growth curves of the human hepatocarcinoma cell lines as measured by the CCK-8 assay. (D) Pearson correlation and simple linear regression analysis of SULT2B1 mRNA levels with cell proliferation (r = 0.931, R^2^ = 0.867, y = 0.9386×−0.2832). (E) Expressions of SULT2B1 mRNA levels in para-tumor and tumor tissues of clinical human hepatocarcinoma samples was detected by qPCR (n = 6). The mRNA levels were normalized to the internal control and represented as the means of results from different samples ± standard error (SE). The PCR products were visualized on a 2% agarose gel containing 5 mg/ml ethidium bromide,GADPH was used as internal control. *represents *P*<0.05 vs. para-tumor tissues.

We also checked the expression of SULT2B1 isoforms in human hepatocarcinoma cell lines Huh-7, Hep3B, SMMC-7721 and BEL-7402 cells. Standard PCR analysis showed that only the SULT2B1b isoform was expressed, although with varying levels (Figure S2). As such, a quantitative PCR analysis was also performed (Fig. 6A) along with an analysis of protein levels (Fig. 6B). Moreover, we assessed different growth ability of those hepatocarcinoma cells by CCK-8 assay (Fig. 6C). Slopes were calculated from the cell growth curve of these four cell lines to reveal their proliferation ability. A regression analysis was performed comparing the relative cell proliferation to the relative SULT2B1 mRNA expression levels. As shown in Fig. 6D, the relative SULT2B1 mRNA expression in human hepatocellular carcinoma cell lines showed a significant and direct correlation with the rate of cell proliferation (r = 0.931, R^2^ = 0.867). As qPCR results indicated that, SULT2B1 mRNA levels in the human hepatocarcinoma tumor tissues showed much higher levels than those of the para-tumor tissues in the clinical samples. Besides, the PCR products were visualized on a 2% agarose gel, which was consistent with the results above (Figure 6E).

### The Suppressive Effects of SULT2B1b Inhibition on Cell Growth, CyclinB1 Expression and Tumorigenicity in Human Hepatocarcinoma Cells

**Figure 7:**
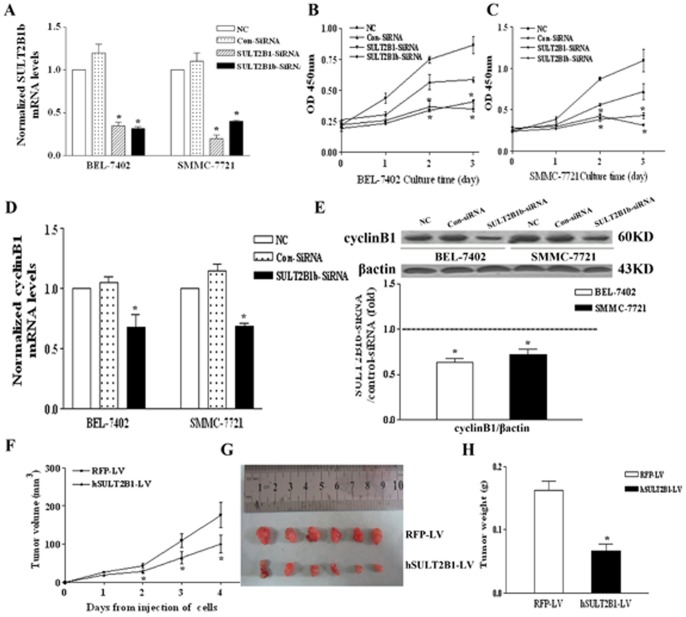
SULT2B1b knock-down suppressed the growth of human hepatocarcinoma cells *in vivo* and *in vitro*. (A) qPCR analysis of SULT2B1b mRNA levels in BEL-7402 and SMMC-7721 cells with SULT2B1 or SULT2B1b-specific siRNA treatment. Cell proliferation rates of BEL-7402 (B) and SMMC-7721 (C) cells treated with SULT2B1 or SULT2B1b-specific siRNA was assessed by CCK-8 assay. (D) cyclinB1 mRNA levels in BEL-7402 and SMMC-7721 cells with SULT2B1b-specific siRNA treatment was detected by qPCR assay. (E) cyclinB1 protein levels in BEL-7402 and SMMC-7721 cells with SULT2B1b-specific siRNA treatment detected by Western-blot analysis, β-actin as internal control. *P<0.05 vs. NC group**.** BEL-7402 cells (1×10^6^) infected with NC-RFP-LV or hSULT2B1-RNAi-LV were injected subcutaneously into right subaxillary region of each nude mouse. The xenograft tumor growth curve (F), representative images (G) and the weights (H) of dissected xenografts were shown. *P<0.05 vs. NC-RFP-LV control group.

Immunocytochemical localization of SULT2B1b in BEL-7402 and SMMC-7721 cells using anti-SULT2B1 antibody revealed a widespread cytoplasmic distribution (Figure S3A,B). Normal rabbit IgG served as negative controls exhibited no green staining. To investigate the effects of SULT2B1b inhibition in human hepatocarcinoma cells, we designed siRNA targeted against SULT2B1 (both SULT2B1a and SULT2B1b isoforms) and specifically against SULT2B1b. qPCR analysis revealed that both SULT2B1 siRNA and SULT2B1b-specific siRNA treatment decreased SULT2B1b mRNA levels significantly in BEL-7402 and SMMC-7721 human hepatocarcinoma cells, as compared with the control siRNA group (Fig. 7A). Moreover, BEL-7402 and SMMC-7721 cells were transiently transfected with siSULT2B1 or siSULT2B1b-specific siRNAs and their cell proliferations assessed by CCK-8 assay. The results show that cell proliferation rates decreased significantly with SULT2B1 inhibition as compared to control cells (Fig. 7B, C).

Further, we detected cyclinB1 expression by qPCR and Western-blot assays. As the results demonstrate, both cyclinB1 mRNA and protein levels decreased significantly with SULT2B1b knock-down in BEL-7402 and SMMC-7721 cells as compared with vector control (Fig. 7D,E). The effect of SULT2B1b interference on tumorigenesis in an *in vivo* xenograft model was further studied. As can be seen in Fig. 7F, SULT2B1b knock-down in BEL-7402 cells significantly suppressed tumor growth *in vivo* as compared with NC-RFP-LV vector control. The tumor size and tumor weight of siSULT2B1b xenografts also was significantly smaller than the with control group (Fig. 7G, H).

## Discussion

In the present study, we demonstrated that the hydroxysterol sulfotransferase, SULT2B1b, promoted proliferation in hepatocellular carcinoma cells both *in vitro* and *in vivo*. Recently, altered expression of SULT2B1b has been demonstrated in hormone-dependent cancers, such as in the breast and prostate [10], [12], [23], [24]. However, the expression and function of SULT2B1b in liver tumors has not been addressed.

Our data suggested that SULT2B1 expressed higher in the human hepatocarcinoma tumor tissues compared to those para-tumor tissues, which suggested that SULT2B1 may play an important role in the hepatocarcinoma cell growth. Additionally, SULT2B1b was the only isoform expressed in both mouse and human hepatocarcinoma cell lines. The localization of SULT2B1b varies in the tissues [7]. He D *et al.* reported that SULT2B1b localized in the nuclei of synchiotrophoblast cells in human term placenta. Likewise, in human T47D and MCF-7 breast cancer cells, SULT2B1b is present both in cytosol and intact nuclei [8]. However, our data showed that SULT2B1b was present in the cytoplasm of hepatocarcinoma cells, but was not detected in the nuclei. There is increasing evidence that supports an association between SULT2B1b and hepatocyte proliferation. Zhang *et al.* reported that both 25HC3S, the biosynthetic product of SULT2B1b, and overexpression of SULT2B1b promoted liver proliferation [17], [25]. Likewise, an increase of SULT2B1 mRNA has also been observed during liver regeneration induced by partial hepatectomy [16]. The correlation between SULT2B1b expression and the proliferative ability of hepatocarcinoma cells was demonstrated. Knock-down of SULT2B1b expression suppressed cell growth in both mouse (Hepa1-6) and human (BEL-7402 and SMMC-7721) hepatocarcinoma cells.

Both the *in vitro* and *in vivo* studies indicated that the inhibition of cell growth by siSULT2B1b was due to increased apoptosis and cell cycle arrest. Hepa1-6 cells showed an imbalance in the expression of pro-apoptotic (also anti-proliferative, FAS) and anti-apoptotic (also pro-proliferative, BCL2 and MYC) proteins after SULT2B1b knock-down, promoting apoptosis and inhibiting proliferation. Our data also suggests that SULT2B1b inhibition significantly increases the apoptosis sensitivity of Hepa1-6 cells to either serum-starvation or TNFα/CHX treatment.

CyclinB1 plays in integral role in many types of cancer. The cyclinB1/Cdk1 complex is the primary regulator of the transition from G_2_ to M phase [26]. Without synthesis of cyclinB1 before the G_2_/M transition, Cdk1 remains inactive, and the cell cannot enter mitosis, resulting in cell cycle arrest at the G_2_ phase [27]. Our data suggests that SULT2B1b inhibition in Hepa1-6 cells can cause G_2_/M phase arrest by decreasing cyclinB1 transcript levels and decreasing its protein stability. Furthermore, the inhibitory effects of siSULT2B1b on the suppressed growth in human hepatocarcinoma cells may also be due to a reduction of cyclinB1 expression.

Based on a nude mice xenograft model using mouse hepatocarcinoma Hepa1-6 and human hepatocarcinoma BEL-7402 cells, both tumor size and tumor weight derived from siSULT2B1b cells was significantly smaller than that of the control group. This inhibitory function of SULT2B1b knock-down on tumor growth may have resulted from increased apoptosis and decreased proliferation. Our results indicated that the proliferation-inhibiting effect of SULT2B1b knock-down is more obvious than an apoptosis-promoting effect *in vivo*.

Zhang *et al.* reported that LXR signaling repression was the main mechanism by which SULT2B1b promotes hepatocyte proliferation *in vitro* [17]. Cook *et al.* detected several isoforms of human SULT (SULT1E1, SULT2A1, and SULT2B1b) were capable of sulfating 24-OHChol, SULT2B1b was the only isoform that formed only 24-OHChol monosulfates which were better inhibitors of LXR activation [28]. This phenomena indicates that SULT2B1b play a role in LXR regulation by sulfating of oxysterols. In our study, whether SULT2B1b knock-down suppressed hepatocellular carcinoma tumorigenicity through LXR pathway should investigated further. However, it is possible that SULT2B1b may promote proliferation directly by upregulating key molecules involved in cell cycle progression. For example, cyclinB1 is a functional target of SULT2B1b knock-down in hepatocarcinoma cells based on *in vitro* or *in vivo* studies. However, the exact mechanism of how SULT2B1b affects cyclinB1 and other important molecules involved in proliferation and apoptosis is not clear and should be further investigated.

### Conclusions

The data demonstrates that the SULT2B1 were comparatively higher in the human hepatocarcinoma tumorous tissues than their adjacent tissues. SULT2B1b promotes the growth of mouse and human hepatocarcinoma cells. Knock-down of SULT2B1b induced cell-cycle arrest and apoptosis, suppressed tumorigenicity in Hepa1-6 cells by up-regulating the expression of FAS, down-regulating the expressions of cyclinB1, BCL2 and MYC *in vitro* and *in vivo*. Our findings suggest a fundamental role of SULT2B1b in HCC and SULT2B1b interference may represent a promising strategy for anti-HCC therapy.

## Supporting Information

Figure S1 **SULT2B1 expression in Hepa1-6 cells.** (A)Western blot analysis of SULT2B1 protein levels in normal C57BL/6 mouse liver, primary mouse hepatocytes, and Hepa1-6 cells. (B) Representative immunofluorescence microscopic analysis of SULT2B1 localization in Hepa1-6 cells. Hepa1-6 NC was represented as negative control which incubated with normal rabbit IgG. Scale bar: 100 µm (C) Expression of mouse SULT2B1a and SULT2B1b isoforms in Hepa1-6 cells transduced with NC-GFP-LV or SULT2B1-RNAi-LV (MOI = 100). Mouse brain tissue was used as mouse SULT2B1a positive control, and β-actin as internal control.
(TIF)

Figure S2 **Endogenous expression of the human SULT2B1a and SULT2B1b isoforms in human hepatocarcinoma cell lines SMMC-7721, BEL-7402, Huh-7 and Hep3B was detected by RT-PCR.** U87 cell line was used as human SULT2B1a positive control, and GADPH as internal control.
(TIF)

Figure S3 **Immunocytochemical localization of SULT2B1b in BEL-7402 and SMMC-7721 cells.** (A and B) Representative immunocytochemical staining of SULT2B1b in BEL-7402 and SMMC-7721 cells. BEL-7402-NC and SMMC-7721-NC were represented as negative control which incubated with normal rabbit IgG. Scale bar: 100 µm.
(TIF)

Table S1 Primers set used for PCR.
(DOC)
