# Supplementary figures and images for: Hydroxysteroid Sulfotransferase SULT2B1b Promotes Hepatocellular Carcinoma Cells Proliferation In Vitro and In Vivo
Source: PLoS One. 2013 Apr 11;8(4):e60853. doi: 10.1371/journal.pone.0060853 (PMC3623875; doi:10.1371/journal.pone.0060853)

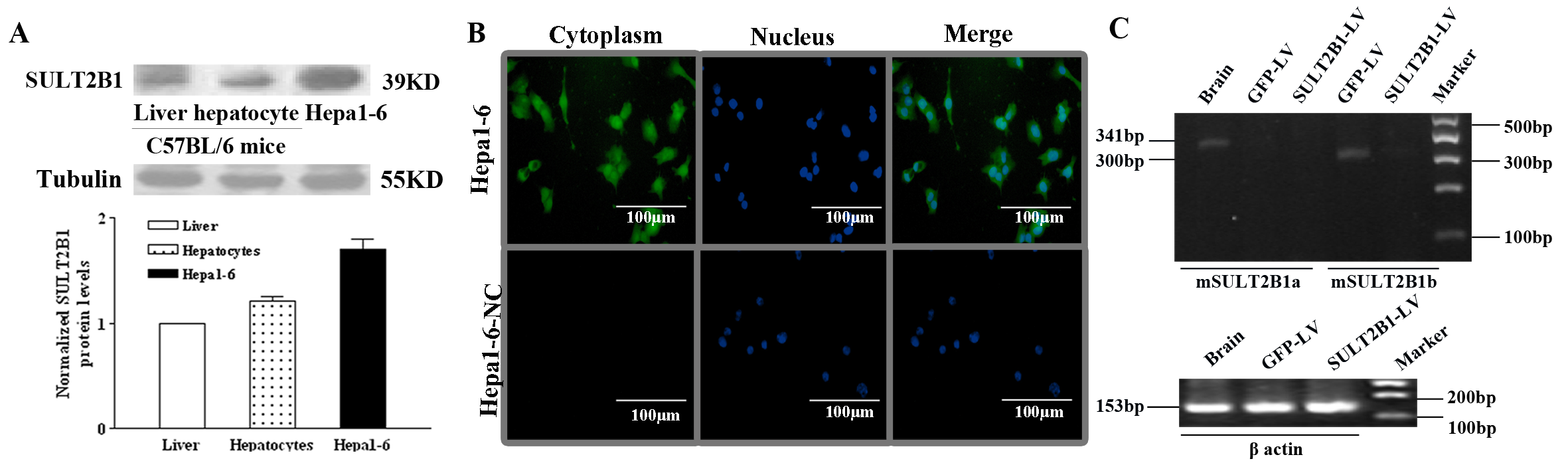

Supplement: Figure S1 — SULT2B1 expression in Hepa1-6 cells. (A)Western blot analysis of SULT2B1 protein levels in normal C57BL/6 mouse liver, primary mouse hepatocytes, and Hepa1-6 cells. (B) Representative immunofluorescence microscopic analysis of SULT2B1 localization in Hepa1-6 cells. Hepa1-6 NC was represented as negative control which incubated with normal rabbit IgG. Scale bar: 100 µm (C) Expression of mouse SULT2B1a and SULT2B1b isoforms in Hepa1-6 cells transduced with NC-GFP-LV or SULT2B1-RNAi-LV (MOI = 100). Mouse brain tissue was used as mouse SULT2B1a positive control, and β-actin as internal control. (TIF) [file pone.0060853.s001.tif]

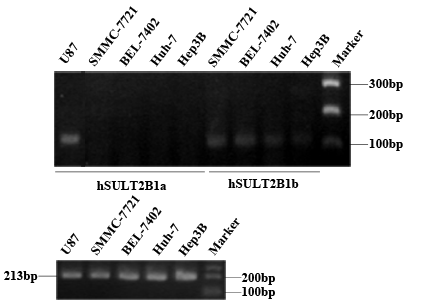

Supplement: Figure S2 — Endogenous expression of the human SULT2B1a and SULT2B1b isoforms in human hepatocarcinoma cell lines SMMC-7721, BEL-7402, Huh-7 and Hep3B was detected by RT-PCR. U87 cell line was used as human SULT2B1a positive control, and GADPH as internal control. (TIF) [file pone.0060853.s002.tif]

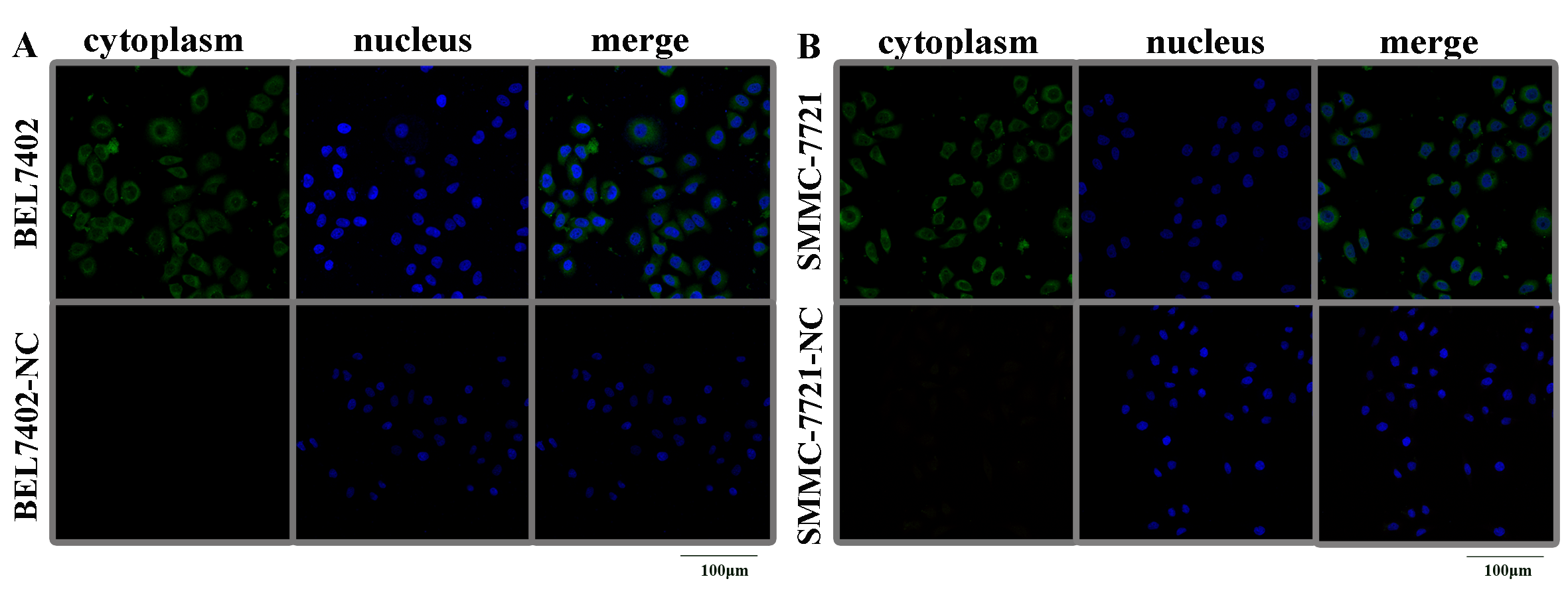

Supplement: Figure S3 — Immunocytochemical localization of SULT2B1b in BEL-7402 and SMMC-7721 cells. (A and B) Representative immunocytochemical staining of SULT2B1b in BEL-7402 and SMMC-7721 cells. BEL-7402-NC and SMMC-7721-NC were represented as negative control which incubated with normal rabbit IgG. Scale bar: 100 µm. (TIF) [file pone.0060853.s003.tif]
