# Supplementary material for: Hydroxysteroid Sulfotransferase SULT2B1b Promotes Hepatocellular Carcinoma Cells Proliferation In Vitro and In Vivo
Source: PLoS One. 2013 Apr 11;8(4):e60853. doi: 10.1371/journal.pone.0060853 (PMC3623875; doi:10.1371/journal.pone.0060853)
Supplement: Table S1 — Primers set used for PCR. (DOC) [file pone.0060853.s004.doc]

**Table S1. Primer sets used for PCR**

| Gene | GenBank Number | Sense Primer (5' - 3') | Antisense Primer (5' - 3') |
| --- | --- | --- | --- |
| mouse SULT2B1a | NM_017465 | ACACTCCTCACTGGCGTGTGAATG | TTGAAGGCGCTTATGATGGTCTCGC |
| mouse SULT2B1b | NM_017465 | GTGGAGCTCGTCTGAGAAAAATGTTTCCG | TTGAAGGCGCTTATGATGGTCTCGC |
| mouse β-actin | NM_007393.3 | GGCTGTATTCCCCTCCATCG | CCAGTTGGTAACAATGCCATGT |
| human SULT2B1a | NM_004605.2 | GTCTCCCCCACCTTTCCA | C ATCTTGGGTGTTCTCCGC |
| human SULT2B1b | NM_177973.1 | ATGACATCTCGGAAATCAGCCA | GCACATCTTGGGTGTTCTCCG |
| human GADPH | NM_002046 | AACGGATTTGGTCGTATTG | GGAAGATGGTGATGGGATT |
